# Characteristics of Acyclovir‐Resistant Herpes Simplex Virus Infection in Hematological Patients

**DOI:** 10.1111/jop.70078

**Published:** 2025-11-12

**Authors:** Reema Shehadeh, Mor Bar Ilan, Sara Dovrat, Ronit Yerushalmi, Michal Tepperberg Oikawa, Evangelia Piperi, Nikolaos G. Nikitakis, Noam Yarom

**Affiliations:** ^1^ Oral Medicine Unit Sheba Medical Center Tel‐Hashomer Israel; ^2^ Oral Medicine Unit, Department of Maxillofacial Surgery Tel Aviv Sourasky Medical Center Tel‐Aviv Israel; ^3^ Central Virology Laboratory Ministry of Health, Sheba Medical Center Tel‐Hashomer Israel; ^4^ Gray Faculty of Medical & Health Sciences Tel‐Aviv University Tel‐Aviv Israel; ^5^ Division of Hematology and Bone Marrow Transplantation Sheba Medical Center Tel‐Hashomer Israel; ^6^ Department of Oral Medicine & Pathology and Hospital Dentistry School of Dentistry, National and Kapodistrian University of Athens (NKUA) Athens Greece; ^7^ Goldschleger School of Dental Medicine, Gray Faculty of Medical & Health Sciences Tel‐Aviv University Tel‐Aviv Israel

**Keywords:** acyclovir prophylaxis, acyclovir resistance, human herpes virus, oral cavity

## Abstract

**Objective:**

Acyclovir‐resistant herpes simplex virus infections pose a significant challenge in immunosuppressed patients. This study aimed to characterize the clinical features, treatment outcomes, and mortality associated with acyclovir‐resistant herpes simplex virus infection.

**Materials and Methods:**

A retrospective analysis was conducted on 18 patients diagnosed with acyclovir‐resistant herpes simplex virus. Diagnosis was confirmed via plaque reduction assay. Data on demographics, underlying conditions, oral and systemic manifestations, management, and outcomes were retrieved and analyzed.

**Results:**

The cohort was comprised of 10 women and eight men with a median age of 41.5 years. Acute myeloid leukemia was the most common underlying medical condition (*n* = 5), and 17 patients had undergone allogeneic hematopoietic cell transplantation. Oral lesions were observed in all 18 cases, primarily on the tongue (*n* = 12) and lips (*n* = 11). Acyclovir resistance was detected after a median of 151.5 days (range: 30–1736 days), after which foscarnet became the primary treatment. Despite therapy, 11 patients died after a median survival of 3 months post‐diagnosis.

**Conclusions:**

Acyclovir‐resistant herpes simplex virus infections are associated with considerable morbidity and mortality in immunocompromised patients. These findings highlight the need for heightened awareness, alternative antiviral therapies, and improved prophylactic strategies to manage these patients more effectively.

**Clinical Relevance:**

Dentists play a key role in detecting acyclovir resistance through persistent oral HSV lesions, emphasizing early recognition, referral, and supportive care.

## 1 Introduction

**FIGURE 1:**
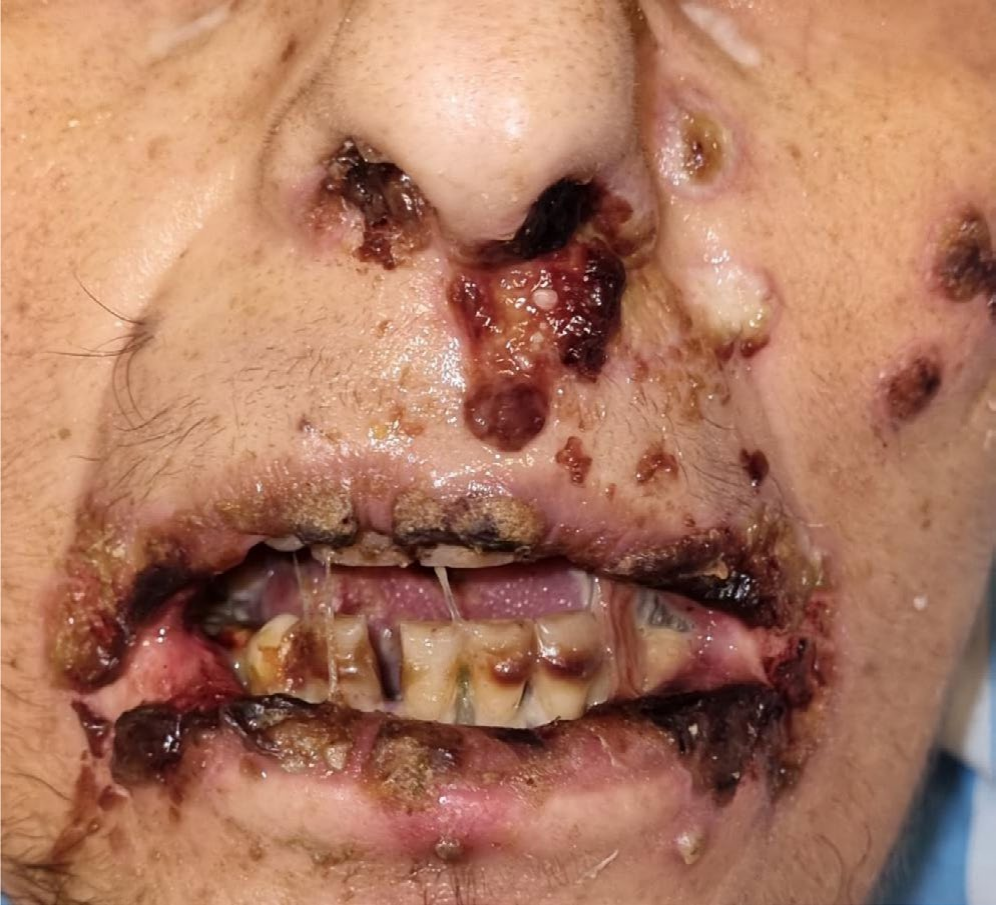
A 57‐year‐old female with oral and perioral lesions associated with acyclovir‐resistant HSV.

Herpes simplex virus (HSV) type 1 (HSV‐1) and type 2 (HSV‐2) rank among the most prevalent human viral pathogens globally, impacting billions of people [1]. HSV‐1 tends to infect the orofacial and ocular regions, and it is typically acquired during infancy or childhood through direct contact with infected oral secretions or active lesions at sites of epithelial damage. HSV‐2 predominantly affects adults and is primarily transmitted through sexual contact, leading to anogenital lesions [2, 3]. The initial HSV‐1 infection presents clinically as primary herpetic gingivostomatitis, while viral reactivation can lead to secondary herpetic stomatitis or, more commonly, to herpes labialis [2, 4, 5]. Both subtypes typically exhibit self‐limiting symptoms, and the treatment objectives are to reduce symptom duration and expedite lesion healing. HSV infections in immunosuppressed individuals, especially those receiving bone marrow or solid organ transplants, as well as HIV‐positive patients, tend to be more severe and even life‐threatening. The herpetic lesions in those patients may be more extensive, have prolonged durations, and recur more frequently, with atypical oral manifestations [6] (Figure 1).

The standard antiviral drugs for treating symptomatic HSV infections are acyclovir (ACV), valacyclovir (Val‐ACV), and famciclovir (FCV) [7]. Immunocompromised patients are more likely to present with viral strains exhibiting resistance due to mutations [8]. The recurrent and widespread use of antivirals, such as ACV and its prodrug, Val‐ACV, has been suggested to contribute to the emergence of resistant strains [9]. Around 95% of ACV‐resistant HSV isolates are associated with mutations in the viral thymidine kinase (TK) enzyme, with only 5% involving mutations in the viral DNA polymerase [10].

Drug resistance can be diagnosed through phenotyping or genotyping, with phenotypic assays measuring virus growth inhibition in the presence of the antiviral drug, while genotypic assays search for specific mutations in the viral genes encoding TK and DNA polymerase [11, 12]. When clinical ACV‐resistant HSV is suspected in patients on oral therapy, the initial recommendation for mild‐to‐moderate disease is to switch to intravenous ACV. Intravenous foscarnet is typically reserved for more severe systemic cases and for those involving the central nervous system [13].

The increased prevalence of ACV‐resistant strains taken together with the adverse clinical consequences of HSV infection in immunocompromised individuals calls for raising the level of awareness of this clinical scenario. This study aims to contribute to the sparse data currently available in publications documenting ACV‐resistant HSV infections and their clinical progression among immunosuppressed patients.

## 2 Methods

### 2.1 Subjects

The medical records of all patients diagnosed with ACV‐resistant HSV at the Sheba Medical Center Virology Laboratory, Tel‐Hashomer, Israel, between 2012 and 2022 were retrieved. All cases were phenotypically diagnosed by plaque reduction assay (PRA) which was requested based upon clinical suspicion of the treating clinician. Data on the demographics, medical background, clinical features of HSV infection, and outcome of anti‐viral treatment were retrieved. Included were patients with clinical features associated with HSV infection and those with a confirmed diagnosis of ACV‐resistant HSV infection. Patients with incomplete clinical information or with borderline PRA results were excluded. Patients without oral lesions associated with HSV infection at the time of HSV sampling, were excluded as well.

The selection of antiviral therapy was based on accepted clinical recommendations [1], the patient's medical status and the response to treatment.

The study was approved by the institutional review board (IRB) of the Sheba Medical Center in accordance with the Declaration of Helsinki and written informed consents were waived for this anonymized analysis.

### 2.2 Methods

The determination of ACV resistance of clinical HSV isolates was done by plaque reduction assay to measure the extent to which various concentrations of acyclovir inhibited the growth of the tested virus in cell culture. The concentration of the drug which resulted in a 50% reduction in plaque formation induced by the viral cytopathic effect versus the no‐drug control established an inhibitory dose (ID‐50) drug concentration reportable value. An ID‐50 between 1 and 3 μg/mL ACV defined an AVC‐sensitive strain while an ID‐50 equal to or above 5 μg/mL defined an ACV‐resistant strain. The assay was performed as follows: oral swabs collected from patients suspected of harboring an acyclovir‐resistant HSV strain were first analyzed for the presence of HSV by real‐time PCR. HSV was then isolated from positive swabs by growing the original sample on Vero cell tissue cultures until the appearance of plaques was visualized by means of a light microscope. The culture medium was then collected and centrifuged at 400 G for 20 min in order to remove cell debris. The clear lysate was considered as the stock virus and it served for further analysis. The stock virus was decimal diluted up to 10 by the seven and 0.2 mL from each dilution was applied to Vero cells in 24‐well plates. The cell medium was replaced with medium containing escalating doses of ACV immediately post‐infection. Untreated wells served as control. The plates underwent fixation and staining at 72 h post infection. ID‐50 of the tested strain was determined based upon the dilution that enabled plaque counting, Laboratory ACV‐sensitive and ACV‐resistant strains were treated similarly and served as controls.

## 3 Results

The dataset included demographic and clinical information for a total of 18 individuals whose average age was 42.1 years (median age 41.5 years, range 9–76 years) (Table 1). The cohort consisted of eight males and 10 females. The most common underlying disease was acute myeloid leukemia (AML), accounting for five cases, followed by four cases each of myelofibrosis and lymphoma. Two patients were diagnosed as having aplastic anemia, two others as having acute lymphoblastic leukemia (ALL), and one patient as having chronic myeloid leukemia (CML). Seventeen of the 18 patients had undergone allogenic hematopoietic cell transplantation (HCT), and the remaining patient had undergone chemotherapy as monotherapy. Prophylactic ACV was administered to 15 patients for up to 24 months (median 4.5 months, average 4.44 months).

**TABLE 1:** Demographics and clinical features of the 18 study patients with ACV‐resistant HSV infection.

Age, years	
Mean	42.1
Median	41.5
Range	9–76
Sex	
Male	*n* = 8 (44.4%)
Female	*n* = 10 (55.6%)
Underling disease	
AML	*n* = 5 (27.8%)
Myelofibrosis	*n* = 4 (22.2%)
Lymphoma	*n* = 4 (22.2%)
Aplastic anemia	*n* = 2 (11.1%)
ALL	*n* = 2 (11.1%)
CML	*n* = 1 (5.6%)
Allogeneic HCT	
Yes	*n* = 17 (94.4%)
No	*n* = 1 (5.6%)
Prophylactic ACV	
Yes	*n* = 15 (83.3%)
No	*n* = 3 (16.7%)
Time between the treatment of the underlying disease and ACV resistance diagnosis, days	
Mean	333
Median	151.5
Range	30–1736
Oral involvement	
Tongue	*n* = 12 (66.6%)
Lips	*n* = 11 (61.1%)
Buccal mucosa	*n* = 6 (33.3%)
Hard palate	*n* = 3 (16.6%)
Soft palate	*n* = 2 (11.1%)
Follow‐up	
Alive	*n* = 7 (38.8%)
Deceased	*n* = 11 (61.11%)

Abbreviations: ACV, acyclovir; ALL, acute lymphoblastic leukemia; AML, acute myeloid leukemia; CML, chronic myeloid leukemia; HCT, allogenic hematopoietic cell transplantation.

The time between treatment of the underlying disease and the detection of ACV resistance varied considerably across patients. The average duration was approximately 333 days (median 151.5 days). The shortest time‐to‐ACV resistance was 30 days, and the longest was 1736 days. Clinical examinations noted lesions on the lining mucosa of all 18 patients, with masticatory mucosal lesions (hard palate) having also formed in three of them. The most involved oral site was the tongue (12 patients), followed by the lips (11 patients), buccal mucosa (six patients), hard palate (three patients), and soft palate (two patients). Details of the specific oral site were not recorded for four patients. Five of the patients had symptoms associated with HSV infection beyond the oral cavity, including the lung (three patients), eyes (two patients), esophagus (two patients), brain (one patient), vagina (one patient), and skin (one patient). HSV was cultured from the bloodstream of three patients and yielded positive results in all 3. In addition, HSV was identified in a vaginal swab (one patient), bronchoalveolar lavage (three patients), esophagus (one patient), eye (one patient), and esophagus (one patient).

Foscarnet was used for treating ACV‐resistant HSV in all patients, often combined with other antivirals (e.g., ganciclovir, valacyclovir, or cidofovir) depending upon clinical findings, and the patient's medical status.

Eleven patients died within less than 6 months (median 3 months) from diagnosis of ACV resistance. While the direct cause of death was not recorded, oral lesions associated with HSV infection were documented in close proximity to the date of death in eight of these patients. The time between the diagnosis of ACV resistance and the last follow‐up of the seven surviving patients ranged from 4 to 9 years (median 5 years).

## 4 Discussion

The present study describes the clinical features of 18 patients diagnosed with ACV‐resistant HSV infection who presented with oral involvement at a tertiary medical center over a 10‐year period. To the best of the authors' knowledge, this is the largest cohort of patients with oral lesions associated with ACV‐resistant HSV infection. The only report in the literature that describes the clinical features of ACV‐resistant HSV infection Ariza‐Heredia et al. [14] includes 18 patients among whom 12 had oral lesions throughout a period of 12 years.

HSV infections are generally self‐limiting. While primary HSV infections and reactivations are typically mild or asymptomatic, they can become severe in immunocompromised individuals, including those undergoing HCT or organ transplantations as well as those who are HIV positive. In those cases, HSV infections may lead to extensive lesions, prolonged illness, and more frequent recurrences, sometimes with atypical manifestations. They are also more likely to develop antiviral‐resistant strains, thereby complicating treatment strategies and increasing mortality risks. All 18 patients in the current report were immunocompromised, with hematologic malignancies, lymphomas, and myelofibrosis being the most common underlying conditions. Similar findings of mostly underlying hematologic malignancies, such as AML, ALL, and CML, were reported by Ariza‐Heredia et al. [14].

The wide range in the time between immunosuppression and resistance development in the current study (from 30 days to over 4 years) underscores the unpredictable nature of ACV resistance in immunocompromised patients. This considerable variation may be influenced by factors such as the intensity of immunosuppression, the presence of co‐infections, and the chronicity of antiviral use [15]. It is also noteworthy that some patients developed resistance relatively early in their ACV prophylactic regimen (within a few months), highlighting the importance of heightened vigilance to provide early detection and timely administration of alternative treatment strategies to prevent the escalation of infection.

Lesions were noted on the lining mucosa of all 18 patients, with only three patients demonstrating coexisting masticatory mucosal lesions (hard palate). This is in contrast to immunocompetent patients, who typically demonstrate HSV lesions on the masticatory mucosa. The most involved oral site was the tongue (12 patients), followed by the lips (11 patients), buccal mucosa (six patients), hard palate (three patients), and soft palate (two patients). Ariza‐Heredia et al. [14] reported that 12 of their 18 patients with ACV‐resistance HSV had coexisting oral/labial manifestations, but those authors did not specify which oral sites were involved.

ACV and its derivatives are considered the gold standard antiviral treatments for preventing and managing HSV infections. However, ACV resistance can develop, particularly in immunocompromised individuals whose impaired cell‐mediated immunity, along with intermittent or prolonged antiviral treatment, is a key factor that contributes to the emergence of resistance. This resistance can lead to severe complications, such as herpetic pneumonia, esophagitis, and meningoencephalitis, potentially making HSV infections life‐threatening [11, 12, 15, 16, 17, 18, 19, 20]. Fifteen of the 18 patients in the current study were receiving prophylactic Val‐ACV or ACV prior to the diagnosis of ACV resistance. Similarly, 17 out of the 18 patients reported by Ariza‐Heredia et al. [14] had received Val‐ACV or parenteral ACV as prophylaxis before their diagnosis. Foscarnet and cidofovir are considered the most effective agents against ACV‐resistant strains due to their activation mechanisms [21]. In the present study, all of the patients were treated with foscarnet as monotherapy or in combination with ganciclovir, valacyclovir, or cidofovir (topical). Foscarnet was also the most prescribed alternative antiviral, often combined with topical agents (e.g., cidofovir or imiquimod) in the cohort described by Ariza‐Heredia et al. [14].

Eleven of the current study patients died within less than 6 months (median 3 months) from ACV‐resistance diagnosis. They all died while on antiviral therapy. The direct cause of death was not definitively known. Oral lesions associated with HSV infection were documented in close proximity to the date of death in 8 of them. Similar findings were reported by Ariza‐Heredia et al. [14]. Nine of their 18 reported patients died within 1 year after the diagnosis of HSV infection, and all without clinical response to antiviral therapy.

A recent in vivo study compared the efficacy of four commercially available mouthwashes against two ACV‐resistant HSV‐1 strains with the aim of determining which product may reduce the shedding of HSV in saliva [6]. All four mouthwashes significantly reduced HSV‐1 infectivity, with essential oil‐based (Listerine Fresh Burst and Listerine Zero) and povidone‐iodine 7.5% (Betadine Gargle) mouthwashes being the most effective, eliminating the virus within 30 s. Chlorhexidine gluconate 0.2% (Hexidyl) was less effective, showing only a 1–2 log reduction in viral viability. Another study evaluated the efficacy of topical cidofovir in managing ACV‐resistant HSV infections in HCT patients [22]. Topical cidofovir was found to effectively reduce HSV‐related oral ulcers and pain in an HCT patient with ACV‐resistant infection without causing nephrotoxicity. These studies support the potential role of topical treatments as an adjuvant treatment for ACV‐resistant HSV infections in immunocompromised patients, especially those at risk of systemic toxicity from traditional antivirals. The possibility that the routine use of the above‐mentioned mouthwashes may prevent the development of ACV‐resistant HSV strains warrants further study.

In conclusion, despite its rarity, ACV‐resistant HSV infection should be considered a potential complication in patients who have undergone HCT. Oral medicine specialists should be alerted to this possibility in order to initiate early management of the condition. The high mortality rate of hematologic patients diagnosed with ACV‐resistant HSV infection emphasizes the need for alternative antiviral options and further clinical research.

## Conflicts of Interest

The authors declare no conflicts of interest.

## Data Availability

The data that support the findings of this study are available on request from the corresponding author. The data is not publicly available due to privacy or ethical restrictions.
